# Efficient production of l-lactic acid by an engineered *Thermoanaerobacterium aotearoense* with broad substrate specificity

**DOI:** 10.1186/1754-6834-6-124

**Published:** 2013-08-28

**Authors:** Xiaofeng Yang, Zhicheng Lai, Chaofeng Lai, Muzi Zhu, Shuang Li, Jufang Wang, Xiaoning Wang

**Affiliations:** 1 Guangdong Key Laboratory of Fermentation and Enzyme Engineering, School of Bioscience and Bioengineering, South China University of Technology, Guangzhou 510006, China; 2 State Key Laboratory of Pulp and Paper Engineering, South China University of Technology, Guangzhou 510640, China; 3 State Key Laboratory of Kidney, the Institute of Life Sciences, Chinese PLA General Hospital, Beijing 100853, China

**Keywords:** l-Lactic acid, Metabolic engineering, Lignocellulosic derived sugars, Xylan, Non-sterilized fermentation

## Abstract

**Background:**

Efficient conversion of lignocellulosic biomass to optically pure lactic acid is a key challenge for the economical production of biodegradable poly-lactic acid. A recently isolated strain, *Thermoanaerobacterium aotearoense* SCUT27, is promising as an efficient lactic acid production bacterium from biomass due to its broad substrate specificity. Additionally, its strictly anaerobic and thermophilic characteristics suppress contamination from other microoragnisms. Herein, we report the significant improvements of concentration and yield in lactic acid production from various lignocellulosic derived sugars, achieved by the carbon flux redirection through homologous recombination in *T. aotearoense* SCUT27.

**Results:**

*T. aotearoense* SCUT27 was engineered to block the acetic acid formation pathway to improve the lactic acid production. The genetic manipulation resulted in 1.8 and 2.1 fold increase of the lactic acid yield using 10 g/L of glucose or 10 g/L of xylose as substrate, respectively. The maximum l-lactic acid yield of 0.93 g/g glucose with an optical purity of 99.3% was obtained by the engineered strain, designated as LA1002, from 50 g/L of substrate, which is very close to the theoretical value (1.0 g/g of glucose). In particular, LA1002 produced lactic acid at an unprecedented concentration up to 3.20 g/L using 10 g/L xylan as the single substrate without any pretreatment after 48 h fermentation. The non-sterilized fermentative production of l-lactic acid was also carried out, achieving values of 44.89 g/L and 0.89 g/g mixed sugar for lactic acid concentration and yield, respectively.

**Conclusions:**

Blocking acetic acid formation pathway in *T. aotearoense* SCUT27 increased l-lactic acid production and yield dramatically. To our best knowledge, this is the best performance of fermentation on lactic acid production using xylan as the sole carbon source, considering the final concentration, yield and fermentation time. In addition, it should be mentioned that the performance of non-sterilized simultaneous fermentation from glucose and xylose was very close to that of normal sterilized cultivation. All these results used the mutant strain, LA1002, indicated that it is a new promising candidate for the effective production of optically pure l-lactic acid from lignocellulosic biomass.

## Background

As an important building block of solvents and substances with biological activities [1,2], lactic acid has attracted increasing attention. For instance, lactic acid can be used to produce biodegradable and biocompatible poly-lactic acid (PLA) [3], which can be used in medical materials as well as in packaging materials. The microbial fermentative production of lactic acid is interesting due to several advantages, *e.g*. low production cost through using cheap raw materials, low production temperature and less energy consumption. In addition, the biological process could produce a desired stereoisomer, optically pure _L_- or _D_-lactic acid, which is a prerequisite for high quality PLA production [4].

To reduce the cost and increase the economy of lactic acid production, utilizing cheap raw materials as resources were extensively investigated, such as molasses, starchy and cellulosic resources. From an economical point of view, lignocellulosic biomass is a potential feedstock for producing lactic acid because they are cheap, abundant and renewable, and do not compete with food [5]. The efficient bioconversion of biomass derived sugars to lactic acid is a key challenge for economically feasible fermentation processes [6]. *Lactobacillus* is the best known commercial strain for lactic acid production due to their high acid tolerance and ability to be genetically engineered for selectively producing optically pure isomers [7,8]. Although lactic acid bacteria (LAB) could produce lactic acid from glucose with a theoretical yield of 100%, most LABs cannot ferment pentose sugars to support growth and metabolize [9]. A few LAB strains metabolize xylose to produce lactic acid via the phosphoketolase (PK) pathway, which exhibits hetero-fermentation of lactic acid and acetic acid and reaches the maximum theoretical lactic acid yield of 1 mol/mol of xylose [9,10]. Abdel-Rahman et al*.*[9] summarized lactic acid production from various types of lignocellulosic biomass materials by LABs through various fermentation models. However, few microorganisms can achieve direct lactic acid fermentation from xylan or dextran. In addition, most LABs produce lactic acid at temperatures of 30–42°C [10], thus medium sterilization is necessary to avoid contamination during fermentation.

Members of *Thermoanaerobacterium* are thermophilic and obligate anaerobic bacteria. They can converse polysaccharide and carbohydrate from lignocellulosic materials to produce primarily l-lactic acid, ethanol, acetic acid, carbon dioxide and hydrogen. Recently, the metabolic pathway has been regulated to achieve high yields of ethanol and hydrogen [11,12]. Argyros et al. [13] reported the co-culture fermentation of an organic acid-deficient engineered *T. saccharolyticum* with a highly cellulolytic organism, *C. thermocellumi*, to produce ethanol from crystalline cellulose.

Herein, we described a genetic alteration in the strain *T. aotearoense* SCUT27 for producing optically pure l-lactic acid via blocking the acetic acid formation pathway. In addition to the ability of metabolizing monosaccharides and disaccharides, the engineered mutant, LA1002, retains the capability of using xylan or dextran as the sole carbon to support cell growth and produce lactic acid in high yield. A non-sterilized anaerobic process to efficiently produce l-lactic acid was also achieved without contamination during fermentation by LA1002. These results indicate that LA1002 could be a promising new optically pure l-lactic acid producer from renewable resources.

## Results and discussion

### Construction of *pta-ack* deficient strains

**Figure 1:**
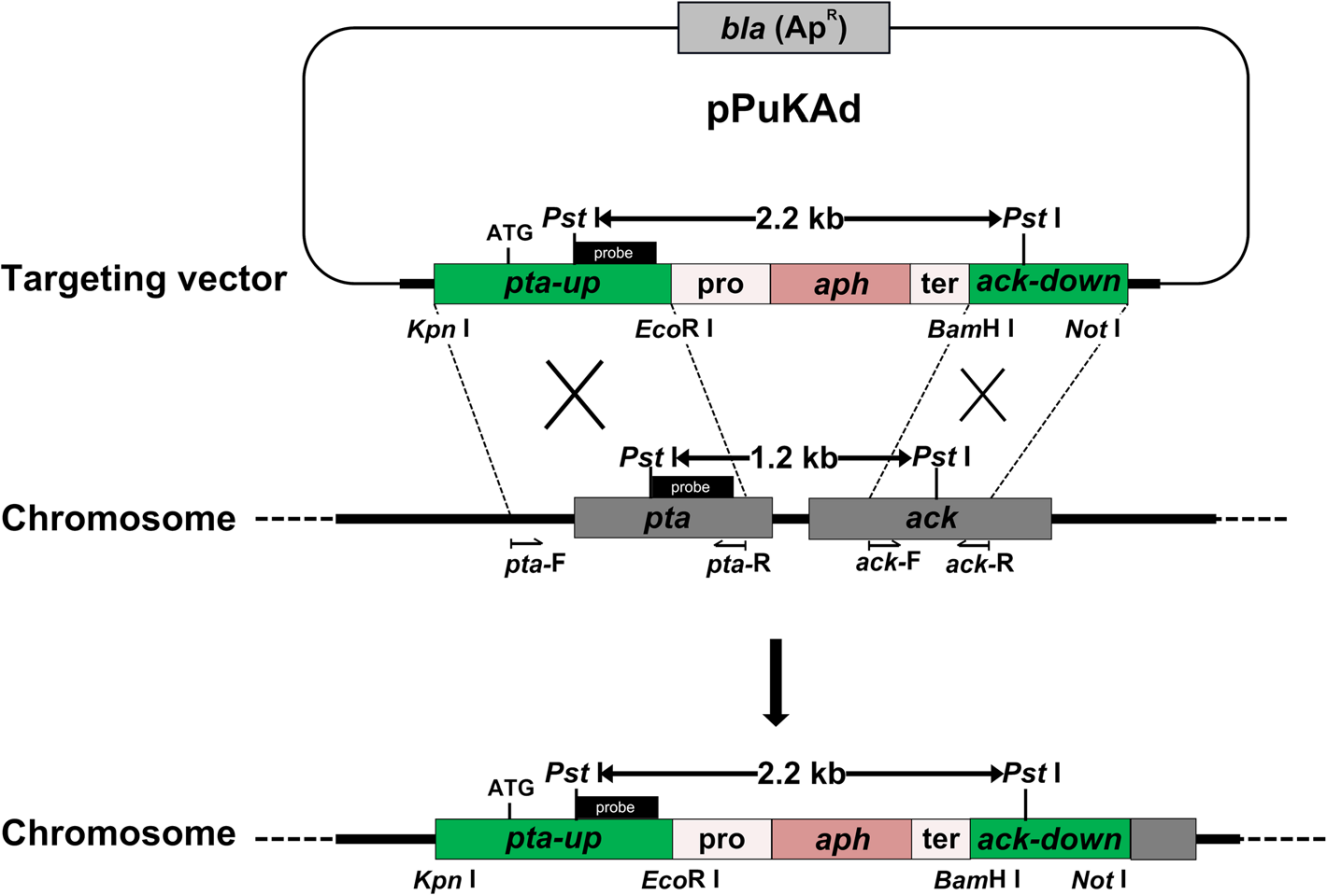
**Schematic diagram of the knockout strategy for the *pta *and *ack *genes.** The *pta-ack* locus on the *T. aotearoense* SCUT 27 chromosome, the pBluescript II SK(+) derived knock out plasmid pPuKAd used to disrupt the *pta-ack* gene locus and the predicted *pta-ack* gene locus after double cross over integration are shown. The endonucleolytic cleavage sites used in the pPuKAd construction are indicated. The location of the probe and the expected sizes of the fragments detected by southern blot analysis of the genomic DNA digested with *Pst* I are shown.

**Figure 2:**
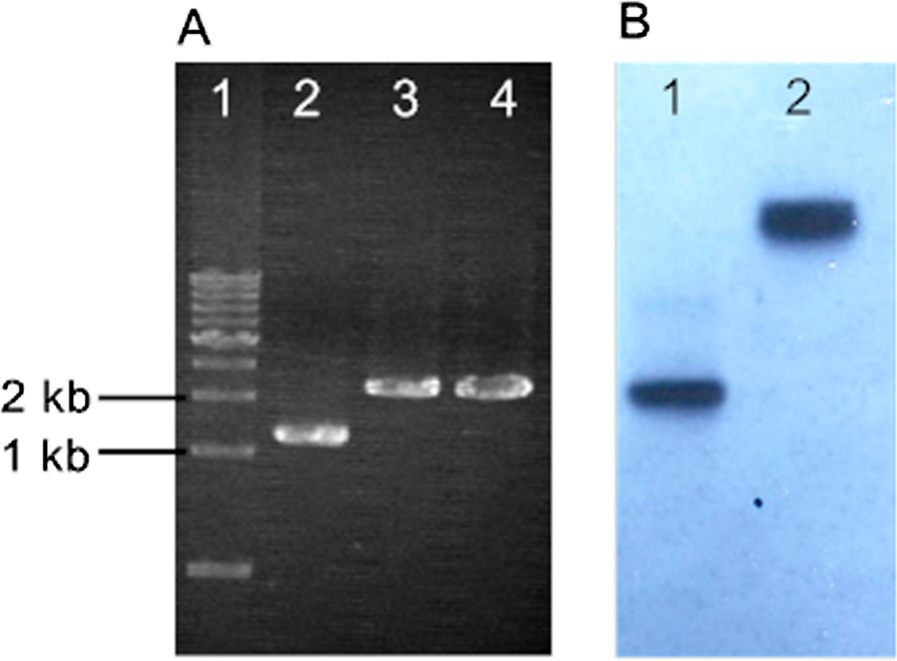
**Screening and confirmation of *pta *and *ack *genes knockout. (A)** polymerase chain reaction (PCR) screening using genomic DNA as template. Lane 1, 1 kb DNA ladder (*T*a*K*a*R*a), Lane 2, SCUT27, Lane 3&4, positive isolates. **(B)** Southern blot analysis of genomic DNA from wild type SCUT27 (Lane 1) and the *pta* and *ack* deletion clones, LA1002 (Lane 2) digested with *Pst* I. The probe with the expected sizes of 486 bp (position shown in Figure 1), hybridized to one 1.2 kb fragment of wild type DNA, and to one 2.2 kb fragment of the mutant DNA.

To block the acetic acid production that consumed the carbon source, the homologous recombinant vector pPuKAd (Figure 1) was transformed into *T. aotearoense* SCUT27 competent cells. Electro-pulsed cells were recovered in liquid medium for 4 h and then plated on agar plates containing 50 μg/mL of kanamycin. After incubation in anaerobic jars at 55°C for 2–3 days, hundreds of colonies grew out. Chromosomal DNA of two picked colonies were extracted. All strains showed positive results analyzed by amplification using primer pair of *pta*-F and *ack*-R. As shown in Figure 2A, negative control with the wild type SCUT27 genomic DNA as template yielded a 2.2 kb fragment. While for the two positive isolates, the amplification obtained 3.3 kb fragments being consistent with the expected size of double-crossover event for chromosomal recombination. The disruption of *pta* and *ack* genes was further confirmed by southern blotting (Figure 2B) using the amplified 486 bp probe. After the genomic DNA digested by *Pst* I, the probe detected a 1.2 kb and a 2.2 kb band for SCUT27 and LA1002, respectively.

### Improved lactic acid production by *T. aotearoense* SCUT27 mutant

**Figure 3:**
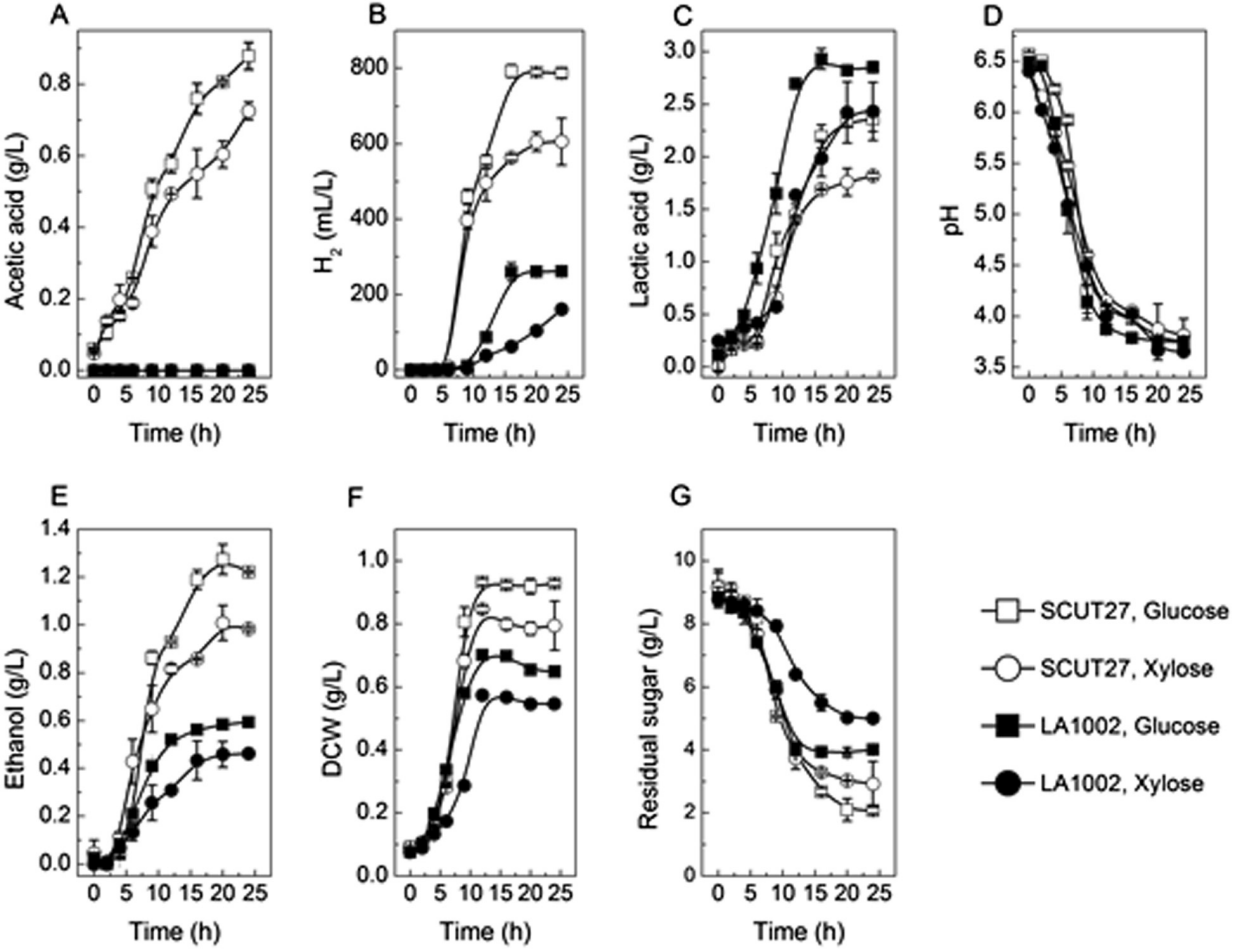
**Fermentation profiles of the SCUT27 and LA1002 strain in 125 mL serum bottles (modified MTC medium containing 10 g/L glucose or xylose as the unique carbon source).** Fermentations were performed in triplicate. All the data were derived from three independent experiments. **(A)** Acetic acid; **(B)** H_2_; **(C)** Lactic acid; **(D)** pH; **(E)** Ethanol; **(F)** DCW; **(G)** Residual sugar.

For the wild and mutant strains, the cell growth and lactic acid production entered into their stationary state after 24 h culturing. So the corresponding metabolites were recorded after 24 h fermentation. During the fermentation in defined media with 10 g/L of glucose or xylose as the carbon source, no acetic acids were detected for the strain LA1002 (Figure 3A). The results further confirmed that the acetic acid formation pathway was disrupted completely in the LA1002. Along with the blocking of acetic acid formation pathway, the hydrogen released by LA1002 was much less than that by SCUT27 (Figure 3B). To test the stability of the kanamycin gene insertion into LA1002 chromosome, the cells were transfer cultured (~100 generation) for 30 days successively in antibiotic-free medium. The cells were plated on media without kanamycin. Genomic DNA was extracted from 20 single colonies of generation 100 and used as template for the insertion amplification with the primers of *pta*-F and *ack*-R. All the LA1002 cells were found containing the 3.3 kb kanamycin insertion fragments, indicating the stability of the phosphotransacetylase and acetate kinase knockout (Additional file 1: Figure S1).

Improving potential production and yielding target products are two of the most anticipated benefits of a metabolic regulation effort. Based on the theoretical analysis of metabolic flux of *T. aotearoense* SCUT27 [11], the carbon flow of mutant strain LA1002 should be redirected to other metabolites, *i.e*. lactic acid and/or ethanol. As shown in Table 1, lactic acid yields by LA1002 from glucose or xylose were increased by 1.79 and 2.07 fold compared to that by SCUT27, respectively. The lactic acid concentration was dramatically enhanced to 2.85 (from glucose) and 2.43 g/L (from xylose) at the end of fermentation by the mutant strain, respectively (Figure 3C). Meanwhile, the maximum lactic acid productivities were improved. It should be noted that the increases of lactic acid yields and specific lactic acid productivities (gram lactic acid/gram cells) from glucose or xylose were almost the same (Table 1), suggesting that the higher lactic acid production was owed to the lactic acid producing ability of single cell. It is interesting that the pH profiles of fermentation broth by SCUT and LA1002 were almost same, at the final pH values of around 3.7 (Figure 3D).

**Table 1:** **Batch fermentation comparison of SCUT27 and LA1002 of *T. aotearoense ***^**a**^

	**Glucose**			**Xylose**		
	**SCUT27**	**LA1002**	**Fold**^**g**^	**SCUT27**	**LA1002**	**Fold**
Carbon recovery^b^	106.02	93.8	-	95.26	99	-
Final pH	3.76	3.77	-	3.81	3.65	-
DCW (g/L)	0.93	0.65	0.70	0.79	0.55	0.69
Consumed sugar (g/L)	7.08	4.83	0.68	6.43	4.23	0.66
*C*_LA_^c^ (g/L)	2.36	2.85	1.21	1.82	2.43	1.34
*Y*_LA_^d^ (g/g)	0.33	0.59	1.79	0.28	0.58	2.07
*P*_LA_^e^ (g/L/h)	0.19	0.29	1.53	0.20	0.22	1.10
*SP*_LA_^f^ (g/g cells)	2.54	4.39	1.73	2.29	4.44	1.94
*C*_EtOH_ (g/L)	1.22	0.59	0.49	0.98	0.46	0.47
*C*_Ac_ (g/L)	0.88	0.00	-	0.73	0.00	-
H_2_ (mL/L)	786.76	261.34	0.33	605.93	161.43	0.27

^a^ Batch fermentation was performed in 125 mL serum bottles containing 50 mL of modified MTC medium, and cultivated with initial pH 6.0 at 55°C for 24 h. The experiments were done on three independent repeats and the standard deviation were not showed in this table for the purposes of simplicity.

^b^ Carbon recovery accounts for the average percentage of carbon recovered in all products and biomass at all time points.

^c^ Lactic acid concentration (g/L).

^d^ Yield of lactic acid produced (g) to consumed sugar (g).

^e^ Lactic acid productivity, calculated as the ratio of lactic acid concentration (g/L) to the fermentation time during the exponential growth.

^f^ Lactic acid specific productivity, in gram lactic acid per gram of cells per hour, were calculated as the ratio of lactic acid concentration to dry cell weight (DCW) during the exponential phase.

^g^ The fold values were calculated by dividing the data of LA1002 by those of SCUT27.

Figure 3E shows that the output of ethanol was reduced to about 50% of that produced by the SCUT27 using glucose or xylose as substrate. Actually, the descended lactic acid production rate during the late fermentation (Figure 3C) was accompanied by the increase of ethanol production (Figure 3E), declination of cell growth (Figure 3F) and decrease in the glucose consumption (Figure 3G). Several hypotheses have been proposed to explain the regulation of lactic acid and glycolytic flux pathway on LABs [14,15], yeasts [16] and other microorganisms [17]. In principle, the redox potential and the energy carriers can be potential modulators of the primary metabolism. Although a lot of modeling strategies were used for the flux analysis, there is no clear scenario to determine the catabolic flux distribution [15]. In this work, the ratio of NADH/NAD^+^ and the pool concentrations of ATP and Pi were all changed through the alteration of acetic acid pathway on the basis of academic analysis. This might account for the drop in ethanol titer produced by LA1002 compared to SCUT27.

As shown in Table 1, the growth of strain LA1002 was inhibited by about 30% of the wild strain SCUT27, measured by DCW (Figure 3F), with almost the same decrease of substrate consumption (Figure 3G). For the acetic acid pathway could generate ATP, the alternation of acetic acid formation resulted in a lower ATP yield by LA1002 than SCUT27. And the lack of energy eventually caused the growth stop [18].

### Effects of carbon sources on the lactic acid production by LA1002

**Figure 4:**
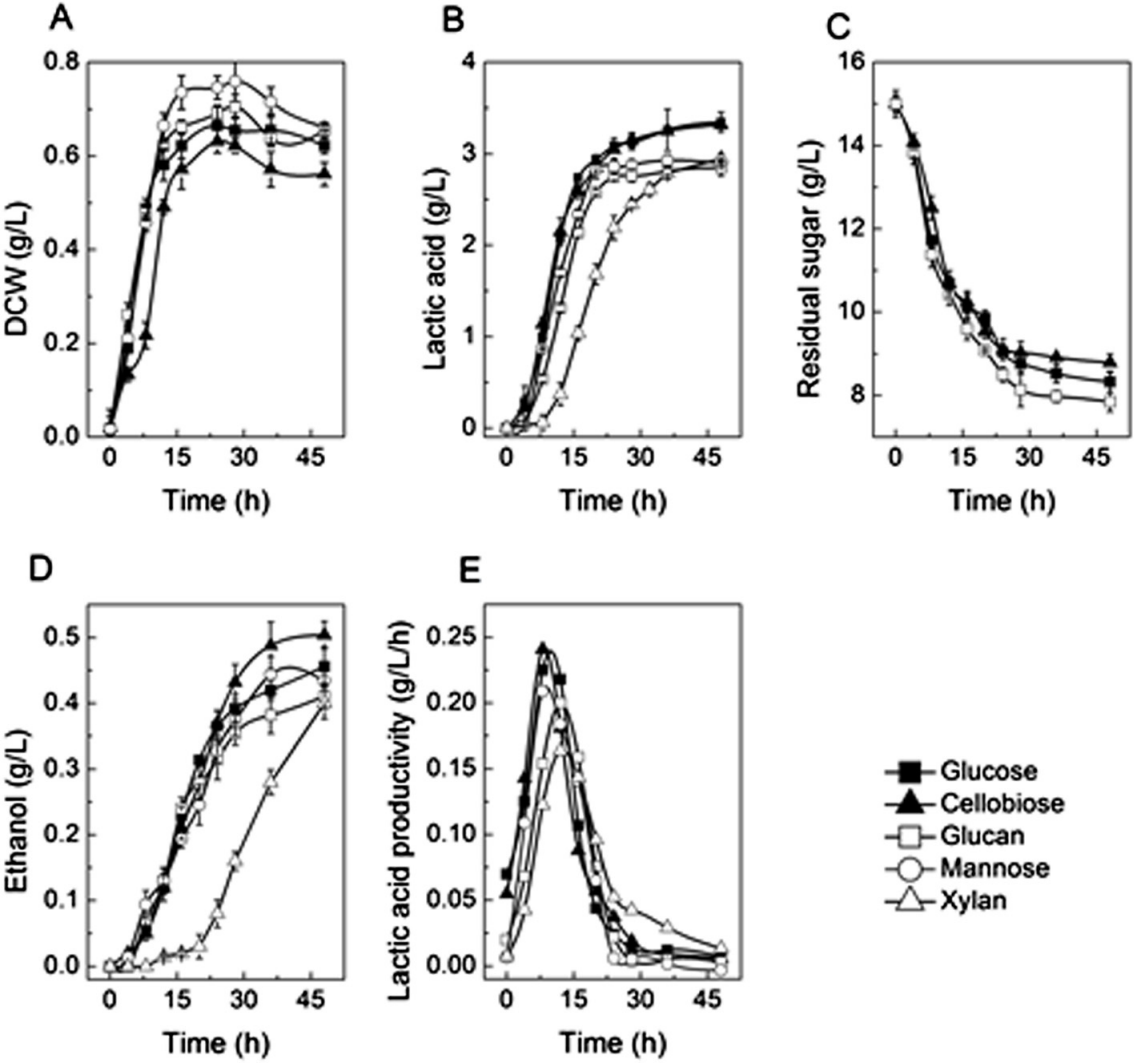
**Time profiles of metabolitics using different sugars as the sole carbon source by LA1002.** The bacterium was cultivated in serum bottles for 24 hours at 55°C with the initial pH of 6.0. Because the fermentation broth was too turbid to determine OD_600_ before xylan degraded, the value of DCW of LA1002 using beechwood xylan as substrate was not measured. And the residual sugar using dextran T110 and xylan as the carbon source were also not recorded. The data were calculated from two independent experiments. **(A)** DCW, **(B)** Lactic acid concentration, **(C)** Residual sugar, **(D)** Ethanol concentration, **(E)** Lactic acid productivity.

In order to evaluate the performance of LA1002 using different sugars derived from lignocellulosic biomass, we conducted fermentation to produce lactic acid using cellobiose, mannose, dextran T110 and beechwood xylan as the sole carbon source. Glucose was used as a control sample. In a 48 h batch fermentation in 125 mL serum bottle containing 50 mL MTC medium, all the sugars (15 g/L) can support the cell growth and lactic acid production by the mutant strain LA1002 (Figure 4A and B). The cell density using benchwood xylan as the sole carbon source was not measured, because the fermentation broth contained some undegraded xylan particles.

The fermentation patterns using the above-mentioned sugars were roughly the same, except that the lag period using xylan as substrate was about double of other situations (Figure 4B,C and D). The fermentation using xylan as carbon source took almost 48 h to reach the maximum lactic acid concentration. While in other cases, it only needed 24–28 h. The maximum lactic acid concentration peaked at 3.38 g/L using cellobiose as a single carbon source followed closely by other sugars, which were all higher than 3.0 g/L (Figure 4B). The final yields of lactic acid were all near 0.52 g/g using glucose, cellobiose and mannose as substrate. The lactic acid productivities during the exponential growth are all around the value of 0.2-0.25 g/L/h, except for that using xylan as single substrate was 0.15 g/L/h (Figure 4E). And the optical purities of l-lactic acid using different carbon source were all higher than 99%. All these results showed that the acetic acid pathway blocked strain LA1002 is capable of using lignocellulos-derived sugars to produce lactic acid.

Recently, some strains capable of utilizing xylose [19-21] and cellobiose [10,22-24] for lactic acid fermentation were reported. Not even that, Okano et al. [25] reported the directed lactic acid fermentation from β-glucan and a cellooligosaccharide by introducing an endoglucanase from a *C. thermocellum* into *L. plantarum* Δ*ldhL1*. The lactic acid concentration reached at 1.47 g/L using 0.2% (w/v) β-glucan as the sole carbon after 33 h cultivation, which was much lower than that obtained by LA1002.

Although a lot of efforts have been put into the lactic acid production using lignocellulos-derived sugars as substrate, rare researches were focused on the xylan utilization. Some wild type and engineered strains have been cultivated with hydrolyzed xylan to produce lactic acid [26,27]. But few strains can metabolize xylan directly without hydrolyzation to produce lactic acid. Ohara et al. [26] reported *Le. lactis* SHO-47 and SHO-54 can utilize xyloheptaose and xylooligosaccharides to produce d-lactic acid, but they still cannot metabolize xylan to support growth. Until recently, an engineered strain of *Lactobacillus brevis* R8 harboring a xylanase gene can produce 1.7 g/L of lactic acid of 1.70 g/L after 4 d of fermentation by using 20 g/L xylan as the main carbon source [28]. By contrast, the lactic acid yield achieved by our constructed strain LA1002 (3.20 g/L) was much higher than that by *L. brevis* R8. Meanwhile, the time required to reach the maximum lactic acid concentration was significantly shortened from 4 d by *L. brevis* R8 to 2 d by LA1002. Besides, to our best known, no reports have examined the lactic acid production using dextran as the sole carbon source. These results indicate that it is very appealing to produce lactic acid through lignocellulose fermentation by LA1002, because the mutant strain is capable of integrating the saccharification of lignocellulose biomass and microbrial fermentation in one pot. It may pave a cost-effective new way to produce lactic acid from lignocellulosic biomass.

### Effects of pH on lactic acid production by LA1002

**Figure 5:**
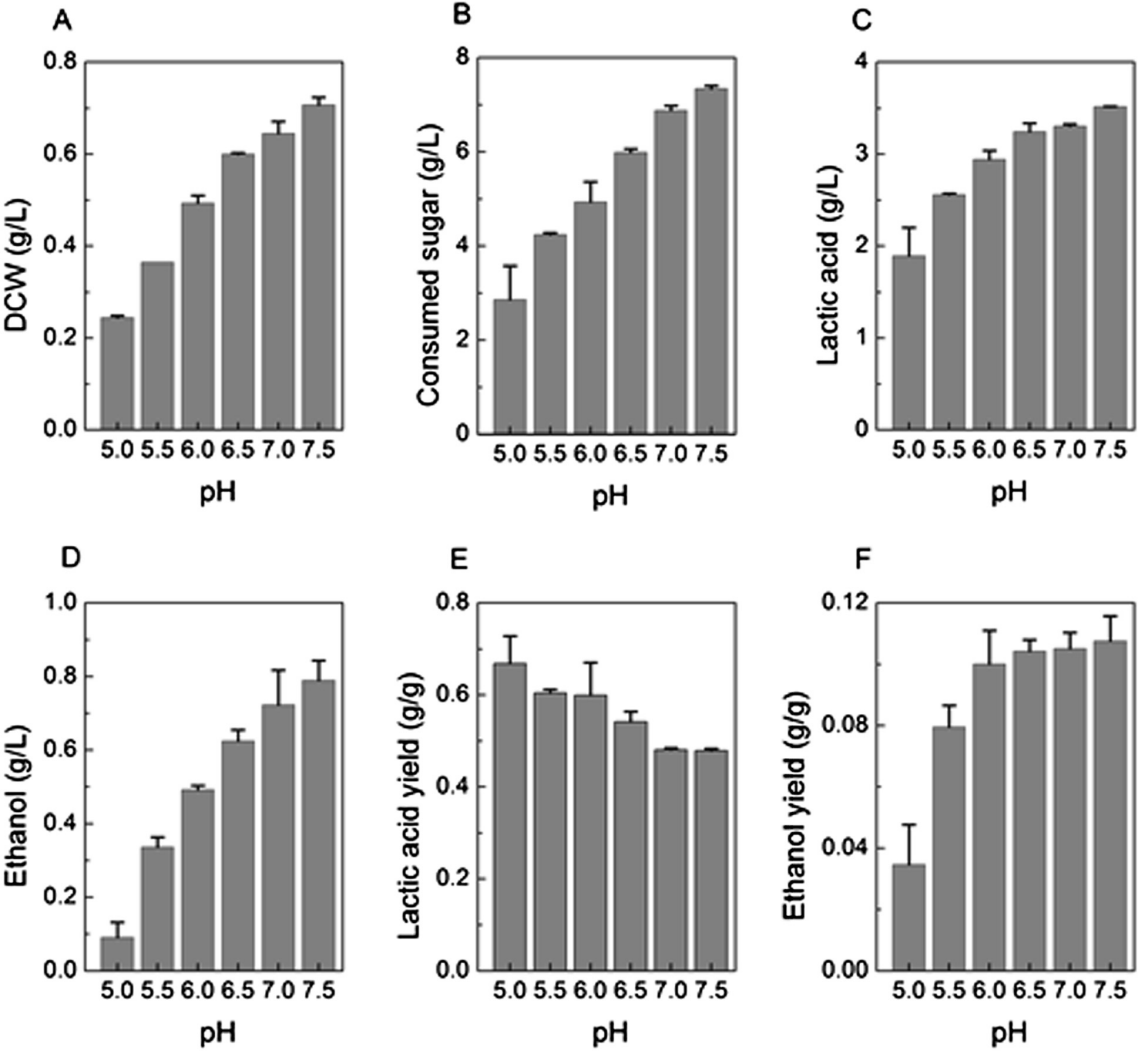
**pH effects on metabolic parameters of lactic acid production by *T. aotearoense *LA1002.** The values are average of three independent experiments and the error bars represent standard deviation. **(A)** DCW; **(B)** Consumed sugar; **(C)** Lactic acid; **(D)** Ethanol; **(E)** Lactic acid yield; **(F)** Ethanol yield.

The extra cellular pH has a big impact on catalytic activities of enzymes and the metabolic flux of microgranisms in fermentation [29,30]. According to our previous work [11], SCUT27 grows bad or could not grow at pH values lower than 5.0 or higher than 7.5. To assess the effect of pH on the lactic acid production by LA1002, we set the initial medium pH from 5.0 to 7.5 with an interval of 0.5 with excess substrate (10 g/L of glucose). After 24 h fermentation, the final culture pH values were all dropped to about 4.0. As shown in Figure 5, the DCW, consumed sugar, ethanol concentration and yield at pH 7.5 were increased by 2.9, 2.6, 9.3 and 3.1 folds using the values at pH 5.0 as benchmark, respectively. However, the lactic acid yield declined slightly as the initial medium pH raised. This indicates that the metabolic flux transits inside cells responding to the changing of extracellular pH value. Higher initial pHs may cause the carbon redirection from lactic acid to cell mass and ethanol formation. On one hand, lactate dehydrogenase is pH sensitive and its activity would be inhibited at increased pH [31]. On the other hand, an increased ratio of NADH/NAD^+^ also suppresses the activity of lactate dehydrogenase, since the NADH/NAD^+^ ratio goes up at higher pHs [29].

Several studies concerned the pH effects on glycolytic flux of different LABs [14,32]. The optimum pH for the lactic acid production and yield by microorganisms is between 5.0 and 7.0, depending on the microorganisms species [29]. Yuwono et al. [33] also concluded that the pH inhibition was competitive for lactic acid concentration and cell growth rate. For the strain LA1002, a pH range of 6.0 and 7.5 was optimal for the lactic acid production and cell growth. Superior results were achieved at pH 6.5 to maximize the lactic acid concentration and yield and to minimize the ethanol formation at the same time.

### pH-controlled high-efficiency lactic acid production by LA1002

Under a non-pH-controlled fermentation in serum bottle, the maximal lactic acid yields were only 0.59 and 0.58 g/g sugar consumed using glucose or xylose as sole carbon source, respectively. The unsatisfactory conversion of sugar to lactic acid may be ascribed to the low pH during the late fermentation (about 4.0 after 24 h fermentation), which has a severe negative effect on cell growth and metabolism. In order to obtain higher lactic acid concentrations and yields, we conducted fermentation assays using 50 g/L of glucose or xylose as carbon source in a 5 L bioreactor with a 3 L working volume at 55°C. All the fermentation processes by LA1002 were carried out at pH 6.5 controlled by the online addition of sodium hydroxide. For all the cultivation, no acetic acid was detected at the end of fermentation with the optical purity of produced l-lactic acid over 99.5%.

First, we carried out lactic acid fermentation using glucose as the carbon source. The sugar consumption, final biomass and the lactic acid production by LA1002 during the pH-controlled fermentation were all increased markedly compared to those acquired by the pH-varied process (Table 2 and Additional file 1: Figure S2). LA1002 completely consumed the total substrate after 36 h cultivation and produced 47.17 g/L of lactic acid with a yield of 0.93 g/g of glucose consumed, which is very close to the theoretical value of 1.0. The maximum specific growth rate and highest volumetric productivity were 0.37 h^-1^ and 2.60 g/L/h, respectively. The dry cell weight was up to 1.69 g/L with only small amounts of ethanol was produced at the concentration ratio of lactic acid to ethanol of 12.89.

**Table 2:** **Fermentation parameters in batch cultivation by LA1002**^**a**^

**Substrate**	**Sterilized**	**Carbon recovery**^**b**^	***μ***_**max**_	**DCW**	**Time**^**c**^	***C***_**LA**_	***Y***_**LA**_	***P***_**LA**_	***C***_**EtOH**_	***C***_**LA**_**/*C***_**EtOH**_
		**(%)**	**(h**^**-1**^**)**	**(g/L)**	**(h)**	**(g/L)**	**(g/g)**	**(g/L/h)**	**(g/L)**	
50 g/L glucose	Yes	107 ± 6	0.37	1.69	36	47.17	0.93	2.60	3.66	12.89
50 g/L xylose	Yes	100 ± 1	0.23	1.78	84	39.72	0.79	0.65	4.63	8.58
25 g/L glucose, 25 g/L xylose	Yes	101 ± 2	0.37	2.10	48	43.56	0.86	1.85	4.12	10.57
25 g/L glucose, 25 g/L xylose	No	98 ± 7	0.32	2.16	60	44.89	0.89	1.26	4.01	11.19

^a^ Batch fermentation was performed in 5 L fermentor containing 3 L medium and cultivated at 55°C controlling pH as 6.5 with 5 M of sodium hydroxide. Data represent the average results from three independent experiments.

^b^ Carbon recovery accounts for the average of percentage of carbon recovered in all products and biomass at all the time points.

^c^ The specified time that strain reached the maximum lactic acid concentration.

Conventionally, calcium carbonate or calcium hydroxide is added to the bioreactor to neutralize the pH during lactic acid fermentation. However,the calcium lactate needs to be acidified with sulfuric acid to convert the salt to lactic acid, which consumes a large amount of sulfuric acid and generates lots of insoluble gypsum (CaSO_4_). Thus alternative neutralizing agents are highly desired to overcome the economic and ecological hurdles of the calcium carbonate neutralization during large-scale lactic acid production [3]. The sodium lactate was the favorite form to the membrane-based lactic acid separation and purification technologies [34,35]. But so far few attempts succeeded in using sodium hydroxide as neutralizing agent, due to the limited tolerance of microorganisms to high concentration of sodium lactate. Qin et al. [34] have reported that a mutant *Bacillus* sp. Na-2 improved resistance against sodium lactate stress, while vigorous agitation and aeration were needed for their process. In this work, the strain LA1002 showed an excellent tolerance to sodium lactate under mild operational conditions, leading a more competitive process of lactic acid fermentation.

A relatively low lactic acid production was observed when used 50 g/L xylose as the sole carbon source. A complete depletion of xylose after 84 h cultivation led to 39.72 g/L lactic acid at a yield of 0.79 g/g of consumed xylose. However, the strain LA1002 showed the ability to utilize xylose in the presence of glucose, though the sugar consumption rate of xylose was lower than that of glucose (Additional file 1: Figure S2B). Most reported microorganisms could not metabolize xylose and glucose simultaneously. Only when the glucose was consumed completely in the medium, xylose began to be fermentated [20,25]. In this study, the simultaneous consumption of xylose and glucose by LA1002 might be ascribed to the leaky expression of xylose transporting and metabolizing genes [25]. The metabolic parameters using mixture sugars as substrate achieved the values of 43.56 g/L, 0.86 g/g, 1.85 g/L/h for lactic acid concentration, yield and productivity, respectively. The maximum growth rate from the mixture substrate, 0.37 h^-1^, was same as the case of pure glucose. The carbon recovery calculation for the time acquired the maximum lactic acid concentration in 5 L fermentor was shown in Additional file 1: Table S1. These results indicate that the LA1002 has the potential to efficiently metabolize hydrolyzed lignocellulosic biomass to produce lactic acid.

### Non-sterilized anaerobic lactic acid production by LA1002

Since *T. aotearoense* LA1002 has a very high cultivation temperature of about 55°C [11], it may enable a non-sterilized anaerobic fermentation and facilitate the separation of lactic acid with the by-product of ethanol. We carried out the non-sterilized fermentation for lactic acid production from a glucose and xylose mixture (Table 2 and Additional file 1: Figure S2). No contamination was observed during the cultivation. The lag time of non-sterilized fermentation was a little longer than that of normal fermentation, due to the growth inhibition to undesired microbes. Even though, the final lactic acid productivity and yield climbed to 44.89 g/L and 0.89 g/g of consumed sugar, respectively, being very close to those of normal sterilized cultivation. Recently, two groups of bacteria have been reported to produce l-lactic acid without sterilization [36-38]. *Bacillus* sp. 2–6 by Qin et al. [37] produced 118.0 g/L of l-lactic acid at the yield of 0.97 g/g glucose under open fermentative conditions. While the thermotolerant strain *B. coagulans* NL01 achieved the maximum lactic acid concentration of about 75 g/L from 100 g/L of xylose after 72 h non-sterilized flask fermentation [38]. To the best of our knowledge, this research is the first to show the simultaneous utilization of glucose and xylose to produce optically pure l-lactic acid at high yields under non-sterilized conditions. All these results further suggested that the engineered LA1002 strain is a promising alternative in the bioconversion of lignocellulosic derived sugars to lactic acid.

## Conclusions

In this study, a thermophilic and anaerobic microorganism of *T. aotearoense* SCUT27 was engineered to produce high concentration l-lactic acid at high yield by blocking the acetic acid formation pathway. No acetic acid by-product was detected during all the fermentation, which significantly facilitate the downstream purification. The mutant LA1002 is able to convert the lignocellulosic sugars, *e.g.* xylose, cellobiose, mannose, dextran and xylan, to optically pure l-lactic acid effectively. More importantly, its thermophilic and anaerobic characteristics allowed for producing lactic acid through a non-sterilized fermentation. Combined with the fermentation merits, *T. aotearoense* LA1002 is encouraging and potentially well-suited for optically pure l-lactic acid production from lignocellulosic biomass in an economic feasible way.

## Methods

### Strains and culture media

*T. aotearoense* SCUT27 used in this study was isolated and maintained in our laboratory [11]. Cells were cultured in modified MTC medium. For the electro-transformation, the electropulsed cells were plated in the modified DSMZ 640 medium by using xylose instead of cellobiose with 2% agar. *Escherichia coli* (*E. coli*) DH5α used for gene cloning was grown in Luria-Bertani (LB) medium supplemented with appropriate antibiotics. When necessary, 50 μg/mL of kanamycin or/and 100 μg/mL of ampicillin were added to the media.

### Gene cloning and suicide vector construction

The bacterial strains, vectors and primers used in this work are listed in Table 3. The *S. faecalis* kanamycin resistance gene 3′5′′-aminoglycoside phosphotransferase of type III (*aph*, Genbank Accession No. V01547) was synthesized by Sangon (Shanghai, China) and inserted between the *Eco*R I and *Bam*H I sites of pBluescript II SK(+) vector (Stratagene, CA, USA), yielding the plasmid pBlue-*aph*. The 1489 bp *aph* sequence employed in this study comprises the 705 bp kanamycin open reading frame (ORF) plus 490 bp upstream of promoter and 294 bp downstream of the transcriptional termination loop. To disrupt the acetic acid formation, the genes encoding phosphotransacetylase (*pta*) and acetate kinase (*ack*) in chromosome from wild type SCUT27 were inserted by *aph* through homologous recombination (Figure 1). Gene fragments of *pta*-up (1179 bp) and *ack*-down (627 bp) are amplified from the genomic DNA of *T. aotearoense* SCUT27 using primer pairs *pta*-F/*pta*-R and *ack*-F/*ack*-R, respectively. Normal PCR amplifications were performed with *pfu* (Stratagene) and *ex*Taq (*T*a*K*a*R*a, Dalian, China) polymerase at the annealing temperatures of 59°C and 55°C for *pta*-up and *ack*-down, respectively. The *pta*-up was first ligated with the plasmid of pBlue-*aph* both doubly digested with *Eco*R I and *Kpn* I, to obtain pBlue-*pta*-*aph*. Then the homologous recombination vector pPuKAd was obtained by subsequently inserting the *ack*-down between the *Bam*H I and *Not* I sites of pBlue-*pta*-*aph*. All restriction enzymes were purchased from *T*a*K*a*R*a.

**Table 3:** Strains, plasmids, and primer sequences used in this study

		
**Strains**		**Source**
*T. aotearoense* SCUT27	Wild type strain	[11]
DH5α	*E. coli* cloning strain, F^-^*endA1 glnV44 thi-1 recA1 relA1 gyrA96 deoR nupG Φ80dlacZ*∆M15 ∆(*lacZYA-argF*)U169, *hsdR17*(*r*_*K*_^-^*m*_*K*_^+^), λ^–^	Invitrogen
LA1002	As SCUT27, but ∆*pta*, ∆*ack*	This study
**Plasmids**	**Description**	**Source**
pBluescript II SK(+)	Standard cloning vector, f1 ori; Amp^R^;	Stratagene
pBlue-*aph*	Derived from pBluescript II SK(+), with kanamycin expression cassette	This study
pBlue-*pta*-*aph*	Derived from pBlue-aph, with partial phosphotransacetylase (*pta*) gene upward of kanamycin gene	This study
pPuKAd	Homologous recombination plasmid derived from pBlue-*pta*-*aph*, with partial acetate kinase (*ack*) gene downward of kanamycin gene	This study
**Primers**	**Sequence 5′ → 3′) **^**a**^	**Application**
*pta*-F	AACTAGGTACCAGCGCTGTACGAAATTGCCACTC	Forward primer for *pta*
*pta*-R	GTACTGAATTCCACCCATTCCTTGTGTTATAGG	Reverse primer for *pta*
*ack*-F	GAGCGGATCCGCATAGAATTAGCTCCACTGC	Forward primer for *ack*
*ack*-R	TGACTGCGGCCGCCGACGCCTCCCATAGCTG	Reverse primer for *ack*
Prob-F	TATTAAGACCTGCATTTCAGAT	Forward primer for hybridization probe
Prob-R	CATTTGCCTTAGCTAACCTC	Reverse primer for hybridization probe

^a^ Underlined nucleotides indicate restriction enzyme sites.

The electro-transformation of *T. aotearoense* SCUT27 was performed as previously reported [11] with modifications during selection on kanamycin. The transformed cells were recovered in liquid modified MTC medium at 50°C for 4 hours, then plated on solid DSMZ 640 medium with 2% of agar containing 50 μg/mL of kanamycin at 50°C for about 3 days. Double homologous recombinants were screened by PCR using genomic DNA as template with the forward primer of *pta*-F and reverse primer of *ack*-R. Southern blotting analysis was carried out to confirm the disruption of *pta* and *ack* genes. By using the primers of Prob-F and Prob-R, the hybridization probe was amplified from the SCUT27 genomic DNA. The obtained knockout mutant was designated as *T. aotearoense* LA1002.

### Serum bottle fermentations

Cultures in 125 mL serum bottle containing 50 mL modified MTC medium with a nitrogen gas headspace and a 10% vol/vol inoculation were carried out at 55°C for the small volume batch fermentation. Samples were removed at specified intervals for determining fermentation parameters of wild type SCUT27 or mutant LA1002. The effects of pH on the cell growth and lactic acid production were studied using 10 g/L glucose as substrate with an initial pH adjusted from 5.0 to 7.5. To study the effect of carbon sources, glucose, xylose, mannose, cellobiose, dextran T110 and beechwood xylan were used as the sole substrate at an initial concentration of 15 g/L for the lactic acid production.

### Bioreactor fermentations

Batch fermentations were performed in a 5.0 L bioreactor (New Brunswick, CT, USA) with a 3 L working volume containing modified MTC medium. The culturing temperature was maintained at 55°C. The agitation speed was kept at 100 rpm. The pH was kept at approximately 6.5 by the automatic addition of 5 M NaOH. Anaerobic conditions were maintained by sparging the medium reservoirs and fermentor with oxygen-free nitrogen for 0.5 to 1 h until the oxygen-indicating dye resazurin became clear. Batch fermentations of the LA1002 strain were performed using 50 g/L of glucose, xylose and glucose/xylose mixture (1:1, w:w). The seed culture was prepared from an overnight culture grown in modified MTC medium containing 5 g/L glucose in serum bottles. Then the inoculum was added into bioreactors at 10% of inoculum volume for lactic acid production. At each sampling time, 5 mL of the cultures were removed and assayed for dry cell weight (DCW), residual sugars and fermentation products.

### Analytical methods

The dry cell weight (DCW) was calculated from the optical density (OD_600_) with a linear correlation factor (DCW (g/L) = 0.0371 + 0.3343 × OD_600_).

Fermentation metabolites and residual sugars contents were determined by high performance liquid chromatography (HPLC) equipped with an Aminex 87H column (Bio-Rad Laboratories, Inc., Hercules, CA) and a refractive index detector. The mobile phase was 5 mM H_2_SO_4_ at a flow rate of 0.6 mL/min. The column temperature was set at 60°C. All samples were passed through 0.22 μm filters before loading. The optical purity of lactic acid was defined as $\frac{\left(L‒\mathrm{lactic}\;\mathrm{acid}\right)‒\left(D‒\mathrm{lactic}\;\mathrm{acid}\right)}{\mathrm{Total}\;\mathrm{lactic}\;\mathrm{acid}}\times \;100\%$. l-lactic acid was measured by SBA-40C lactate biosensor analyzer (The Academy of Science in Shandong Province, China), and the total lactic acid was determined by HPLC. Carbon balance calculations were based on the previously reported equation [11].

5 mL of hydrogen was drawn out from serum bottle sealed with rubber and thin aluminium sheet tightly, then 1 mL was injected into gas chromatography (Fuli 9790, China) immediately for hydrogen quantity measurement. The gas chromatography equipped with a TDX-01 column and an AE electric insulating oil analysis column, a thermal conductivity detector (TCD). The oven temperature was isothermally set at 60°C. The calculation details can be acquired from our previous work [11].

## Abbreviations

pta: Phosphotransacetylase; ack: acetate kinase; aph: 3′5″-Aminoglycoside phosphotransferase of type III; DCW: Dry cell weight; PCR: Polymerase chain reaction; HPLC: High-performance liquid chromatography.

## Competing interests

The authors declare that they have no competing interests.

## Authors’ contributions

XY designed and carried out the genetic and fermentation experiment. ZL and MZ participated in the fermentation and data analysis. CL carried out the genetic manipulation. SL participated in design of the study and data analysis, coordination of the work, and writing of the manuscript. JW conceived of the study and helped to draft the manuscript, coordination of the work. XW helped to revise the manuscript. All authors read and approved the final manuscript.

## Supplementary Material

Additional file 1: Figure S1 Genetic stability detection of LA1002 by PCR using *pta*-F and *ack*-R as primers with genomic DNA as template. M: 1 kb DNA ladder (TaKaRa), 1-20, different single colonies of LA1002 (generation 100), P: LA1002 (generation 1) as the positive control, N: SCUT27 as the negative control. **Figure S2.** Fermentation of single substrate or mixtures of glucose/xylose (1:1, *w*:*w*) by LA1002 in 5 L bioreactor using sterilized or non-sterilized culture medium. (A) DCW, (B) Residual sugars, (C) Lactic acid concentration, (D) Lactic acid production rate. Panel (A), (C) and (D), ▲glucose, ▼xylose, □ mixture of glucose/xylose, ○ non-sterilized mixture glucose/xylose. Panel (B), ▲glucose,▼ xylose, □ residual glucose in the mixture, ○ residual xylose in the mixture, ■residual glucose in the non-sterilized mixture, ●residual xylose in the non-sterilized mixture. **Table S1.** Carbon recovery calculation in batch cultivation by LA1002^a^. Click here for file
