# Supplementary material for: Efficient production of l-lactic acid by an engineered Thermoanaerobacterium aotearoense with broad substrate specificity
Source: Biotechnol Biofuels. 2013 Aug 28;6:124. doi: 10.1186/1754-6834-6-124 (PMC3766646; doi:10.1186/1754-6834-6-124)
Supplement: Additional file 1: Figure S1 — Genetic stability detection of LA1002 by PCR using pta-F and ack-R as primers with genomic DNA as template. M: 1 kb DNA ladder (TaKaRa), 1-20, different single colonies of LA1002 (generation 100), P: LA1002 (generation 1) as the positive control, N: SCUT27 as the negative control. Figure S2. Fermentation of single substrate or mixtures of glucose/xylose (1:1, w:w) by LA1002 in 5 L bioreactor using sterilized or non-sterilized culture medium. (A) DCW, (B) Residual sugars, (C) Lactic acid concentration, (D) Lactic acid production rate. Panel (A), (C) and (D), ▲glucose, ▼xylose, □ mixture of glucose/xylose, ○ non-sterilized mixture glucose/xylose. Panel (B), ▲glucose,▼ xylose, □ residual glucose in the mixture, ○ residual xylose in the mixture, ■residual glucose in the non-sterilized mixture, ●residual xylose in the non-sterilized mixture. Table S1. Carbon recovery calculation in batch cultivation by LA1002a. [file 1754-6834-6-124-S1.docx]

Additional file

For

**Efficient production of l-lactic acid by an engineered *Thermoanaerobacterium aotearoense* with broad substrate specificity**

Xiaofeng Yang^1^, Zhicheng Lai^1^, Chaofeng Lai^1^, Zhicheng Lai^1^, Muzi Zhu^1^, Shuang Li^1,2,^*, Jufang Wang^2,^*, Xiaoning Wang^3^

^a^ *Guangdong Key Laboratory of Fermentation and Enzyme Engineering, School of Bioscience and Bioengineering, South China University of Technology, Guangzhou 510006, China*

^b^ *State Key Laboratory of Pulp and Paper Engineering, South China University of Technology, Guangzhou510640, China*

^c^ *State Key Laboratory of Kidney, the Institute of Life Sciences, Chinese PLA General Hospital, Beijing 100853,China*

**Correspondence author:*

Shuang Li

Guangzhou Higher Education Mega Center, Panyu District, Guangzhou, P.R. China, 510006

Tel: +86 20 3938 0629

Fax: +86 20 3938 0629

E-mail: [shuangli@scut.edu.cn](mailto:shuangli@scut.edu.cn)

Jufang Wang

Guangzhou Higher Education Mega Center, Panyu District, Guangzhou, P.R. China, 510006

Tel: +86 20-3938 0626

Fax: +86 20-3938 0626

E-mail: jufwang@scut.edu.cn

*E-mail address of other authors:*

Xiaofeng Yang: [xfyang1987@163.com](mailto:xfyang1987@163.com)

Zhicheng Lai: [tindltailor@foxmail.com](mailto:tindltailor@foxmail.com)

Chaofeng Lai: laichaofeng@126.com

Muzi Zhu: [814820458@qq.com](mailto:814820458@qq.com)

Xiaoning Wang: [xnwang88@163.com](mailto:xnwang88@163.com)


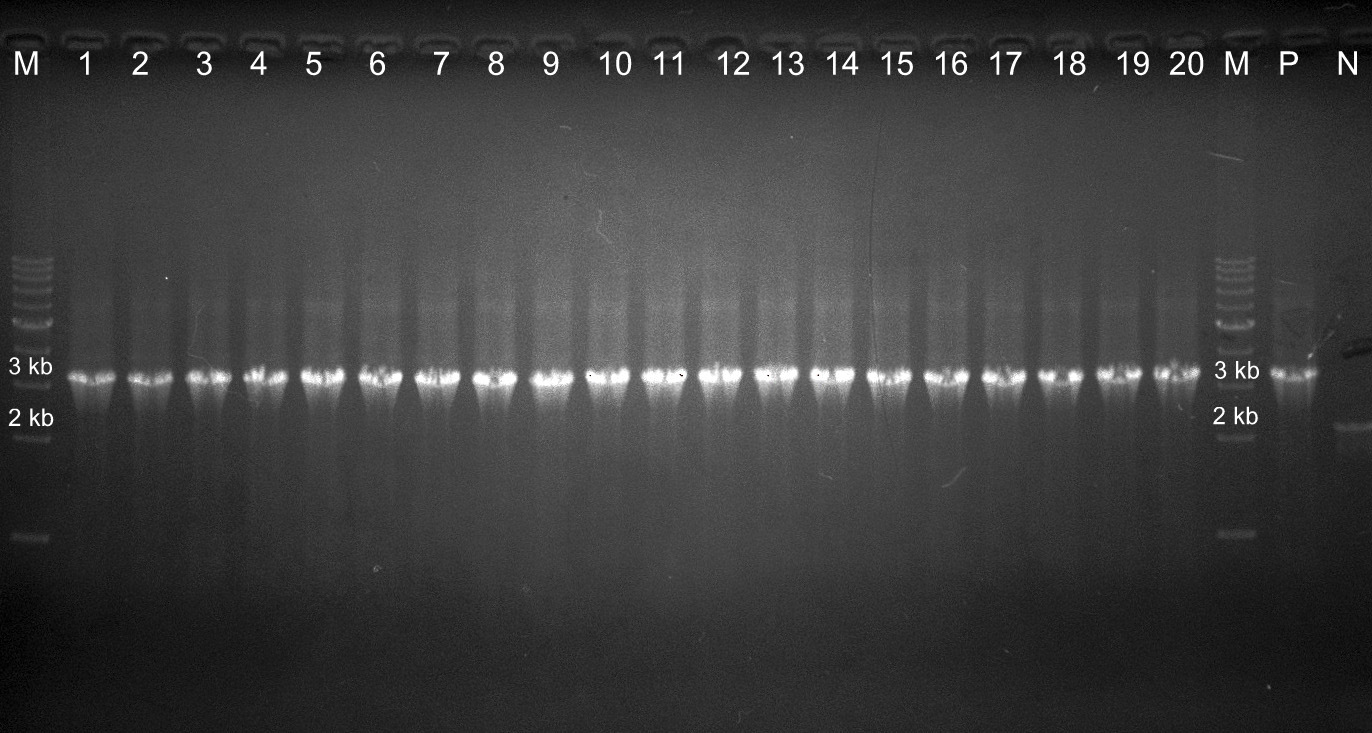


Figure S1. Genetic stability detection of LA1002 by PCR using *pta*-F and *ack*-R as primers with genomic DNA as template. M: 1 kb DNA ladder (TaKaRa), 1-20, different single colonies of LA1002 (generation 100), P: LA1002 (generation 1) as the positive control, N: SCUT27 as the negative control.


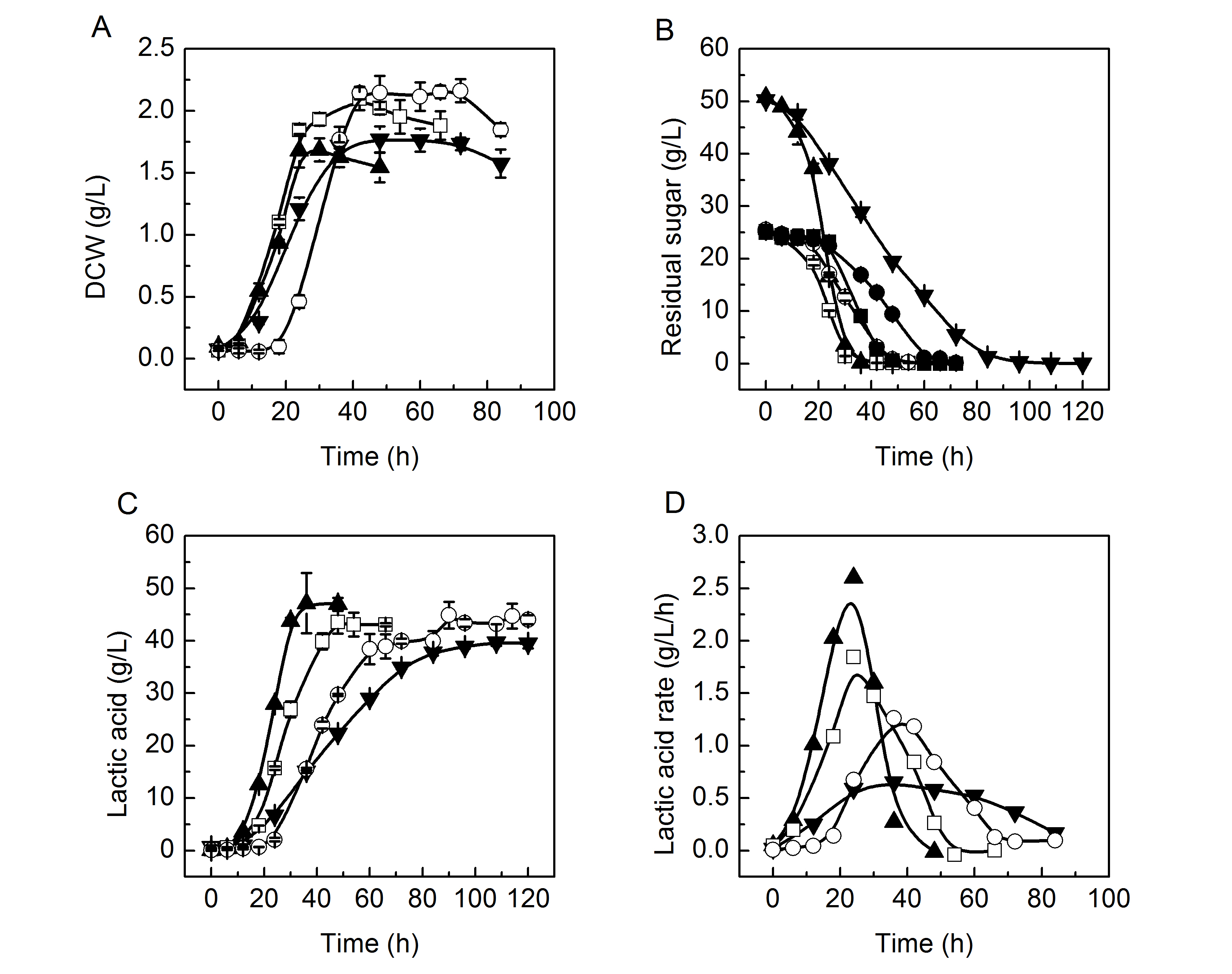


**Figure S2.** Fermentation of single substrate or mixtures of glucose/xylose (1:1, *w*:*w*) by LA1002 in 5 L bioreactor using sterilized or non-sterilized culture medium. (A) DCW, (B) Residual sugars, (C) Lactic acid concentration, (D) Lactic acid production rate. Panel (A), (C) and (D), ▲glucose, ▼xylose, □ mixture of glucose/xylose, ○ non-sterilized mixture glucose/xylose. Panel (B), ▲glucose,▼ xylose, □ residual glucose in the mixture, ○ residual xylose in the mixture, ■residual glucose in the non-sterilized mixture, ●residual xylose in the non-sterilized mixture.

**Table S1. Carbon recovery calculation in batch cultivation by LA1002^a^**

| Substrate | Sterilized | Input^b^ | |  | Output^c^ | | | | | | | | | Carbon recovery^e^ |
| --- | --- | --- | --- | --- | --- | --- | --- | --- | --- | --- | --- | --- | --- | --- |
|  |  | Sugar | Carbon |  | Lactic acid | | Ethanol | | CO_2_ | | DCW | | Total |  |
|  |  | g/L | g/L |  | g/L | %^d^ | g/L | % | g/L | % | g/L | % | g/L |  |
| 50 g/L glucose | Yes | 50.82 | 20.33 |  | 18.87 | 88.78 | 0.90 | 4.25 | 0.69 | 3.23 | 0.79 | 3.74 | 21.25 | 1.05 |
| 50 g/L xylose | Yes | 50.25 | 20.10 |  | 15.89 | 79.44 | 2.41 | 12.04 | 0.87 | 4.34 | 0.84 | 4.18 | 20.00 | 0.99 |
| 25 g/L glucose, 25 g/L xylose | Yes | 50.72 | 20.29 |  | 17.42 | 81.70 | 2.14 | 10.05 | 0.77 | 3.62 | 0.99 | 4.63 | 21.33 | 1.03 |
| 25 g/L glucose, 25 g/L xylose | No | 50.05 | 20.02 |  | 17.96 | 82.34 | 2.09 | 9.56 | 0.75 | 3.45 | 1.02 | 4.66 | 21.81 | 1.08 |

^a^ Data represent the point that fermentation achieved maximum lactic acid concentration in 5 L fermentor containing 3 L medium.

^b^ The input carbon (g/L) was calculated as 0.4 Sugar (g/L), where the sugar can be glucose, xylose and the mixture of glucose and xylose.

^c^ The output carbon (g/L) was calculated as 0.4 Sugar + 0.4 Lactic acid + 0.52 Ethanol + 0.26 CO_2_ + 0.47 DCW. For the sugar was consumed completely, the table did not show the data of output sugar. The biomass was estimated using the general empirical formula for cell composition of CH_2_N_0.25_O_0.5_. Carbon dioxide was accounted for by stoichiometric correlations to ethanol, 1mol CO_2_ will be released when 1 mol ethanol produced. And the carbon contained in extracellular protein and yeast extract of medium was not considered in the carbon balance calculation.

^d^ Percentages of lactic acid, ethanol, carbon dioxide and biomass to total output carbon.

^e^ The carbon recovery was calculated as the ratio of total output carbon to input carbon.
